# Identification of Key Amino Acid Residues Modulating Intracellular and *In vitro* Microcin E492 Amyloid Formation

**DOI:** 10.3389/fmicb.2016.00035

**Published:** 2016-01-28

**Authors:** Paulina Aguilera, Andrés Marcoleta, Pablo Lobos-Ruiz, Rocío Arranz, José M. Valpuesta, Octavio Monasterio, Rosalba Lagos

**Affiliations:** ^1^Laboratorio de Biología Estructural y Molecular, Departamento de Biología, Facultad de Ciencias, Universidad de ChileSantiago, Chile; ^2^Centro Nacional de Biotecnología, Consejo Superior de Investigaciones CientíficasMadrid, Spain

**Keywords:** microcin E492, intracellular amyloids, gatekeeper residues, protein aggregation, inclusion bodies

## Abstract

Microcin E492 (MccE492) is a pore-forming bacteriocin produced and exported by *Klebsiella pneumoniae* RYC492. Besides its antibacterial activity, excreted MccE492 can form amyloid fibrils *in vivo* as well as *in vitro*. It has been proposed that bacterial amyloids can be functional playing a biological role, and in the particular case of MccE492 it would control the antibacterial activity. MccE492 amyloid fibril’s morphology and formation kinetics *in vitro* have been well-characterized, however, it is not known which amino acid residues determine its amyloidogenic propensity, nor if it forms intracellular amyloid inclusions as has been reported for other bacterial amyloids. In this work we found the conditions in which MccE492 forms intracellular amyloids in *Escherichia coli* cells, that were visualized as round-shaped inclusion bodies recognized by two amyloidophilic probes, 2-4′-methylaminophenyl benzothiazole and thioflavin-S. We used this property to perform a flow cytometry-based assay to evaluate the aggregation propensity of MccE492 mutants, that were designed using an *in silico* prediction of putative aggregation hotspots. We established that the predicted amino acid residues 54–63, effectively act as a pro-amyloidogenic stretch. As in the case of other amyloidogenic proteins, this region presented two gatekeeper residues (P57 and P59), which disfavor both intracellular and *in vitro* MccE492 amyloid formation, preventing an uncontrolled aggregation. Mutants in each of these gatekeeper residues showed faster *in vitro* aggregation and bactericidal inactivation kinetics, and the two mutants were accumulated as dense amyloid inclusions in more than 80% of *E. coli* cells expressing these variants. In contrast, the MccE492 mutant lacking residues 54–63 showed a significantly lower intracellular aggregation propensity and slower *in vitro* polymerization kinetics. Electron microscopy analysis of the amyloids formed *in vitro* by these mutants revealed that, although with different efficiency, all formed fibrils morphologically similar to wild-type MccE492. The physiological implication of MccE492 intracellular amyloid formation is probably similar to the inactivation process observed for extracellular amyloids, and could be used as a mean of sequestering potentially toxic species inside the cell when this bacteriocin is produced in large amounts.

## Introduction

Amyloid fibrils are highly organized protein aggregates with a unique quaternary structure named cross-β, consisting of protein monomers assembled into intermolecular hydrogen bonded β-strands placed perpendicularly to the fibril axis (Chiti and Dobson, 2006; Fowler et al., 2007). The unit of the cross-β spine is a β-sheet bilayer with side chains within the bilayer forming a tight “steric zipper” (Sawaya et al., 2007; Fitzpatrick et al., 2013). Amyloids share chemical properties such as specific binding of probes (Nilsson, 2004) as well as denaturation and proteolysis resistance. Traditionally, amyloid-fibrils formation has been related to neurodegenerative pathologies such as Alzheimer’s, Parkinson’s, and Huntington’s disease. However, several examples of amyloids playing a biological role have appeared during the last years. These “functional amyloids” have been described in many organisms: from bacteria and fungi, to insects, fish, and mammals (Fowler et al., 2007). The first reported example of a bacterial functional amyloid was curli, a well-studied type of extracellular amyloid fibrils produced by *Escherichia coli* and *Salmonella* that participate in biofilm formation, host cell adhesion and invasion (Chapman et al., 2002; Wang et al., 2006). Other examples include TasA produced by the gram-positive bacteria *Bacillus subtilis*, where the amyloid fibrils also participate in biofilm development (Romero et al., 2010), and the filamentous bacteria *Streptomyces coelicolor*, in which the aerial hyphae growth is mediated by amyloid structures formed by the chaplin proteins (Claessen et al., 2003).

Most examples of bacterial amyloids are extracellular. One exceptional and interesting case is RepA, the replication initiator protein of *Pseudomonas* plasmid pPS10. In the presence of short dsDNA oligonucleotides, the RepA-WH1 domain forms amyloid fibrils *in vitro* (Giraldo, 2007). The expression in *E. coli* of the hyper-amyloidogenic domain variant A31V fused to a red fluorescent protein, led to the accumulation of amyloid inclusions into the cytosol. Remarkably, these amyloid inclusions were transmitted vertically to the progeny and bacteria carrying them showed decreased cell fitness, constituting a bacterial proteinopathy (Fernández-Tresguerres et al., 2010). Regarding intracellular amyloid formation, it has been found that mammalian amyloid proteins expressed in *E. coli* form intracellular amyloid aggregates as well (Dasari et al., 2011; Espargaró et al., 2012). Moreover, when analyzing the ultrastructure of inclusion bodies formed by a growing number of proteins (even those not formally defined as amyloidogenic), it was noticed that they have an amyloid-like cross-β structure (Carrió et al., 2005; Wang et al., 2008a). Additionally, it was demonstrated that the co-expression of the yeast amyloidogenic proteins Sup35 and New1 in *E. coli* cells leads to the formation of cytoplasmic inclusions with amyloid properties, and its formation is correlated with the propagation of the prionic/amyloid form of Sup35, termed [PSI^+^] (Yuan et al., 2014).

It is now evident that proteins capable of forming amyloid structures are quite diverse, and that the amyloidogenesis phenomenon is more ubiquitous than originally thought. Thus, it has been suggested that amyloid formation is a generic property of the polypeptide chain and not a feature of a small number of proteins (Stefani and Dobson, 2003). Moreover, it has been shown that the propensity to form amyloid fibrils depends on specific regions or residues within the polypeptide chain. The “pro-amyloidogenic regions” or “aggregation hotspots” initiate or favor the conformational transition into the cross-β assembly and often consist of 5–15 adjacent hydrophobic residues of low net charge and a high tendency to form β-strands (Beerten et al., 2012). In addition, aggregation propensity can also be modulated by a group of residues called *gatekeepers*, located near or within the pro-amyloidogenic region. These residues prevent an uncontrolled aggregation disfavoring β-structure formation, and are usually charged residues or prolines (Rousseau et al., 2006; Beerten et al., 2012).

Microcin E492 is a low-molecular-weight channel-forming bacteriocin produced by *Klebsiella pneumoniae* RYC492 (de Lorenzo, 1984; Lagos et al., 1993, 2009). It is found in two forms: unmodified (7,887 Da) and post-translationally modified in its C-terminal end by the covalent linkage of glycosylated salmochelin derivatives of different molecular masses (Thomas et al., 2004). This modification is required for antibacterial activity, since toxin uptake by the target cells depends on the recognition of the salmochelin-like moiety by the outer membrane catecholate siderophore receptors FepA, Fiu, and Cir (Strahsburger et al., 2005). Once in the periplasm, MccE492 exerts its toxic activity through the formation of pores in the cytoplasmic membrane and the consequent membrane potential dissipation (de Lorenzo and Pugsley, 1985; Lagos et al., 1993). One salient feature of MccE492 is its ability to form amyloid fibrils, which was observed *in vitro* and *in vivo* in the extracellular space, and was associated with the loss of antibacterial activity (Bieler et al., 2005; Marcoleta et al., 2013). Hence, amyloid formation was proposed as a mechanism of antibacterial inactivation. Even though MccE492 amyloid fibril’s morphology and formation kinetics *in vitro* have been well-characterized (Bieler et al., 2005; Arranz et al., 2012; Marcoleta et al., 2013), it is not known if MccE492, as in the case of RepA, forms amyloid *in vivo* inside the cell. Besides the influence of the post-translational modification on retarding the kinetics of amyloid formation (Marcoleta et al., 2013), there are no studies on how the primary structure, specifically which amino acid residues, determines its amyloidogenic propensity. In this work we report the conditions in which MccE492 forms intracellular amyloid inclusions. We used this phenomenon to perform a flow cytometry-based screen for MccE492 mutants with altered aggregation propensity, establishing which regions and residues act as pro-amyloidogenic regions or as gatekeepers, promoting or disfavoring both intracellular and *in vitro* amyloid formation. Additionally, our results suggest that there is a factor encoded in the MccE492 genetic cluster that may act promoting intracellular MccE492 amyloidogenesis. The promotion of cytoplasmic MccE492 amyloid formation suggests that this phenomenon may have a physiological implication as a mechanism of capturing and inactivating an excess of toxic species inside the bacteria.

## Materials and Methods

### Bacterial Strain and Plasmids

**FIGURE 1:**
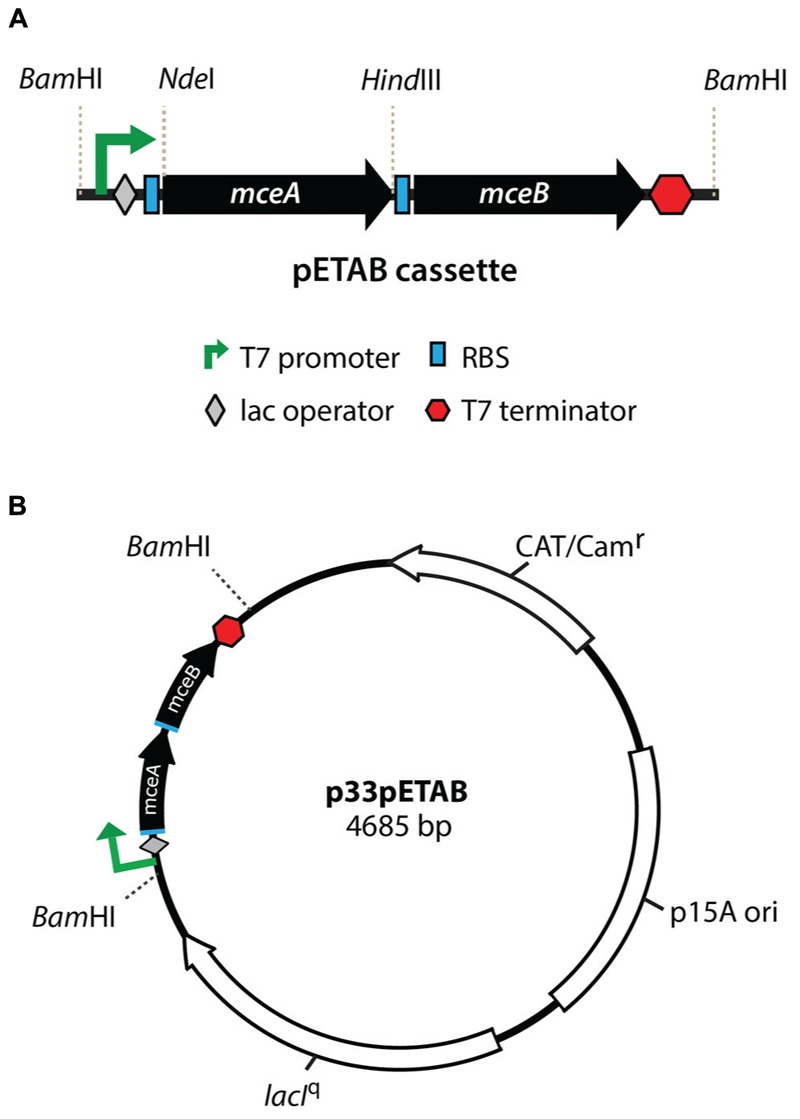
**Properties of the pETAB cassette for regulated expression of MccE492.** Genes *mceAB* were cloned under a T7 promoter with further regulation by *lac* operator **(A)**. A consensus ribosome binding site (RBS) was included upstream of each coding region. This cassette was introduced in the *BamH*I site of p33AM, generating p33pETAB **(B)** that expresses constitutively *lacI^q^*, to repress expression from the T7 promoter in the absence of IPTG. Different versions of the *mceA* genes can be replaced in this construction using the *Nde*I*/Hind*III restriction fragment.

The bacterial strain and plasmids used in this work are shown in **Table 1**. A vector for tight expression of MccE492 and mutagenized derivatives was designed. This construct harbors both *mceA* (MccE492) and *mceB* (immunity protein) genes under the control of a T7 promoter, a *lac* operator and a T7 terminator (pETAB cassette, **Figure 1A**). The expression of the immunity is required since MccE492 expression is toxic in the absence of this protein (Bieler et al., 2006). It is important to note that naturally, *mceB* and *mceA* (in this order) form a single transcriptional unit where the last 23 nucleotides of *mceB* and the first 23 nucleotides of *mceA* overlap. Results from our laboratory indicate that there is an internal promoter inside the *mceB* coding region that contributes to the expression of *mceA*. For this reason we decided to invert the order of the genes in the cassette, and to insert a consensus ribosome binding site to each coding region in order to achieve a comparable expression of both genes, and to avoid leaky transcription of *mceA* from the *mceB* internal promoter. Additionally, to allow the easy replacement of the *mceA* gene by distinct variants and the cloning of the pETAB cassette, *Nde*I/*Hind*III and *Bam*HI sites were conveniently included. pETAB was cloned in the p33AM plasmid backbone generating p33pETAB, which harbors a copy of the *lacI*^q^ repressor gene and a p15A origin (**Figure 1B**). Two different plasmids compatible with the p33pETAB system, carrying the whole or part of the MccE492 production cluster were used in this work: pMccE492 and np220. pMccE492 comprises all the necessary components for active microcin production, i.e., the structural and immunity genes and the genes involved in post-translational modification, export, regulation, and others of unknown function, in the same disposition as in *K. pneumoniae* RYC492 chromosome, as it is depicted in Lagos et al. (2009). The expression of this plasmid results in the production of active MccE492. Meanwhile, np220 is a plasmid used in our lab as a background to produce and export post-translationally modified MccE492 (Mercado et al., 2008) expressed from a compatible plasmid, such as p33pETAB and pBAML. np220 encodes all the components necessary for MccE492 activity, such as immunity, export and post-translational modification, with a gene disposition that it is not exactly the same than in *K. pneumoniae* RYC492. In this plasmid the structural gene *mceA* is interrupted by a Tn5 insertion, thus np220 by itself is unable to produce MccE492 (Lagos et al., 2001). Another plasmid used in this work is pBAML, which only harbors the structural *mceA* and the immunity *mceB* genes with their natural promoter and in the configuration described above for *K. pneumoniae* RYC492. The expression of pBAML as well as p33pETAB in the absence of np220 results in the production of unmodified and non-exported MccE492.

**Table 1:** Bacterial strain and plasmids used in this work.

Bacterial strain or plasmid	Relevant genotype or features	Source
***Escherichia coli* strain**		
BL21-AI	F- *omp*T *hsd*SB (rB-mB-) *gal dcm ara*B::*T7RNAP tet*A	Invitrogen
**Plasmids**		
pHC79	ColE1 cosmid derivative. Amp^r^ Tet^r^	Hohn and Collins, 1980
pMccE492	Whole MccE492 production system, pHC79 derived. Amp^r^	González, 2011
np220	Whole MccE492 production system, but *mceA::Tn5*. Amp^r^ Kan^r^	Mercado et al., 2008
pBAML	MccE492 structural (*mceA*) and immunity (*mceB*) genes. Cam^r^	Lagos R. laboratory collection
p33AM	*lacIq.* Allows the regulation of the pET system. P15A origin, Cam^r^	Lagos R. laboratory collection
p33pETAB	p33AM derivative. Contains the pETAB cassette (*mceA* and *mceB* under the control of the T7 promoter) and *lacIq.* Cam^r^	This work
N16A	p33pETAB derivative. *mceA* was mutagenized replacing asparagine 16 by alanine. Cam^r^	This work
P26A	p33pETAB derivative. *mceA* was mutagenized replacing proline 26 by alanine. Cam^r^	This work
Δ18–35	p33pETAB derivative. *mceA* was mutagenized deleting amino acids 18–35. Cam^r^	This work
P53A	p33pETAB derivative. *mceA* was mutagenized replacing proline 53 by alanine. Cam^r^	This work
N55A	p33pETAB derivative. *mceA* was mutagenized replacing asparagine 55 by alanine. Cam^r^	This work
P57A	p33pETAB derivative. *mceA* was mutagenized replacing proline 57 by alanine. Cam^r^	This work
P59A	p33pETAB derivative. *mceA* was mutagenized replacing proline 59 by alanine. Cam^r^	This work
P64A	p33pETAB derivative. *mceA* was mutagenized replacing proline 64 by alanine. Cam^r^	This work
Δ54–63	p33pETAB derivative. *mceA* was mutagenized deleting amino acids 54–63. Cam^r^	This work

### Growth Conditions

Bacterial growth was performed incubating with shaking (180–220 rpm) at 37°C. For confocal microscopy analysis cells carrying MccE492 producing systems were grown in M9 minimal medium supplemented with citrate 0.2% and glucose 0.2% for 48 h (late stationary phase). For intracellular aggregation propensity determinations, cells carrying p33pETAB variants with or without np220 (this plasmid allows the modification and exportation of MccE492) were grown in LB medium. Antibiotics were used in the following concentrations: ampicillin (Amp) 100 μg/ml, kanamycin (Kan) 50 μg/ml, and chloramphenicol (Cam) 50 μg/ml.

### MccE492 Induction Assay

MccE492 induction assay in the p33pETAB/BL21-AI system was performed as follows: 15 ml of LB (with the corresponding antibiotic) were inoculated with 1:20 dilution of fresh overnight *E. coli* BL21-AI p33pETAB culture. The cells were grown at 37°C with shaking at 180 rpm until early exponential phase was reached. At this point, 10 ml of culture were split in two (5 ml each): one aliquot was induced with 0.2% arabinose and 1 mM IPTG, and the other was left untreated. After 6 h, aliquots of induced and non-induced cells were collected to continue with the protein extraction protocol or with the fixation and staining procedure.

### Total Protein Extraction

For total protein extraction, cells were centrifuged at 12,900 × *g* for 10 min. The pellet was re-suspended in PBS buffer, incubated at 95°C for 20 min and centrifuged at 12,900 × *g* for 15 min at 4°C. 400 μl of the supernatant were collected and 100 μl of cold TCA were added. The mixture was incubated 10 min at 4°C and then centrifuged at 12,900 × *g* for 15 min at 4°C. The supernatant was discarded and the pellet was washed twice with 200 μl of chilled acetone. Finally, acetone was evaporated and the pellet was stored at -80°C.

### SDS-PAGE and Immunoblotting

Sodium dodecyl sulfate-polyacrylamide gel electrophoresis (SDS-PAGE) was performed as described by Schägger and von Jagow (1987). Nitrocellulose membranes (Millipore) were used for immunoblot transfer (1 h, 350 mA, using chilled 25 mM Tris-HCl, 190 mM glycine, 20% methanol, as the transfer buffer). MccE492 was detected with a polyclonal antibody prepared in rabbit against the last 20 amino acids of the protein (antiserum dilution, 1:1,000) and with a goat anti-rabbit alkaline phosphatase-conjugated secondary antibody (dilution 1:5,000). The alkaline phosphatase colorimetric reaction was performed as described by Marcoleta et al. (2013). The membrane was washed with FAL buffer (100 mM Tris-HCl [pH 9.5], 100 mM NaCl, 5 mM MgCl_2_) and incubated in 10 ml of a mixture of BCIP (5-bromo-4-chloro-3′-indolylphosphate *p*-toluidine salt) and NBT (nitroblue tetrazolium chloride; 0.3 and 0.15 mg/ml in FAL buffer, respectively), until an optimal signal was observed.

### Cell Fixation and Staining for Confocal Microscopy

Five hundred μl of bacterial culture were washed twice with the same volume of PBS buffer by centrifuging at 1,100 × *g* for 4 min at room temperature, fixed by suspending in 250 μl of 4% PFA in PBS and incubating for 30 min at room temperature. BTA-1 staining was performed as described (Fernández-Tresguerres et al., 2010). After fixation, cells were washed twice with 250 μl of PBS buffer by centrifuging at 1,100 × *g* for 4 min at room temperature, re-suspended in 1 mM BTA-1 prepared in 100% ethanol, and incubated for 30 min at room temperature. Finally, cells were washed twice with PBS. ThS staining was carried out based on the protocol of Espargaró et al. (2012) with some modifications: 500 μl of bacterial culture were centrifuged at 1,100 × *g* for 4 min at room temperature, the supernatant was discarded, and the bacterial pellet was washed twice with 500 μl of PBS buffer. Cells were suspended in 250 μl of 0.05% (w/v) ThS in 12.5% ethanol and incubated for 1 h at room temperature. We found that ThS staining in the presence of 12.5% ethanol, instead of PBS as originally described, leads to a smaller number of false negative cells. After staining, cells were washed three times with 250 μl of PBS buffer. Stained cells were mounted over a glass slide covered by layer of 1% agarose in PBS.

### Confocal Microscopy

Microscopic observations were performed using an LSM 710 (Zeiss) confocal microscope, with a 63x/NA 1.40 oil immersion objective. ThS fluorescence was excited using a 488 nm argon laser and the emission was registered in a range from 410 to 520 nm. BTA-1 fluorescence was excited with a 405 nm laser diode and the emission was registered between 493 and 552 nm. Images were digitally captured with ZEN 2012 and analyzed with ImageJ software (Schneider et al., 2012).

### Flow Cytometry

Thioflavin-S staining for flow cytometry analysis was carried out in the same way as described for confocal microscopy, but starting with unfixed cells. Flow cytometry measurements were performed using a BD FACSCanto^TM^ II flow cytometer. Cells stained with ThS were first gated by forward scatter (FSC) and side scatter (SCC) signals, and then analyzed for ThS fluorescence by exciting at 405 nm and registering the emission at 510/550 nm.

### MccE492 Purification

MccE492 (wild-type or mutant) was purified from culture supernatants of *E. coli* BL21-AI cells carrying each p33pETAB variant along with np220, to allow MccE492 processing and export. Briefly, 4 L of M9 medium supplemented with 0.2% citrate and 0.1% glucose were inoculated with 1:1000 dilution of fresh overnight culture and grown at 37°C with shaking at 180 rpm for 6 h (until exponential phase). At this point, 0.2% arabinose and 1 mM IPTG were added. After 12–14 h, the supernatant was collected by centrifugation and filtered through a 0.22 μm polyethersulfone membrane. The cell-free medium was incubated with 25 g of previously ACN-activated Bondapak C18 resin (Waters) at 4°C for 2 h. The resin was filtered by negative pressure through a Buchner funnel, washed with 200 ml of 40% methanol, then with 200 ml of 25% ACN, and finally eluted with a 30–100% ACN stepwise gradient. MccE492-enriched fractions were dialyzed twice for 2 h against 40 volumes of 5 mM Tris (pH 8.5) and then lyophilized and stored at -20°C.

### *In vitro* MccE492 Amyloid Formation followed by Congo Red Binding Assay

An appropriate amount of lyophilized MccE492 powder was dissolved in 5 mM Tris (pH 8.5), and centrifuged for 30 min at 16,000 × *g* to eliminate preformed aggregates. The supernatant was diluted to 0.4 mg/ml in the aggregation buffer (100 mM PIPES-NaOH, 0.5 M NaCl [pH 6.5]) and incubated with agitation (800 rpm) during the entire assay. Amyloid formation was quantified by the diminution of free Congo red using the following procedure: at the indicated times, MccE492 samples were incubated at 37°C for 15 min with 33 μM Congo red and centrifuged at 16,000 × *g* for 40 min at 4°C. The supernatant absorbance was registered at 490 nm, and the free Congo red fraction was determined as the ratio between the absorbance registered at each time and the absorbance at time zero.

### Determination of Soluble MccE492 During the Aggregation Curve

To visualize soluble MccE492 during the aggregation assays, aliquots of the samples taken at different times were collected and centrifuged at 16,000 × *g* for 40 min at 4°C. The supernatant was recovered and the remaining soluble protein was detected by SDS-PAGE and immunoblotting.

### MccE492 Antibacterial Activity Determination

MccE492 activity was determined by the critical dilution method (Mayr-Harting et al., 1972). At the indicated times, aliquots from the aggregation assays were collected and serially diluted in sterile nanopure water. Three μl of each dilution were seeded onto a lawn of a sensitive *E. coli* strain, prepared by mixing 0.3 ml of the *E. coli* culture with 3 ml soft agar and overlaying the resulting mixture onto LB plates. MccE492 antibacterial activity was detected by the formation of growth inhibition halos, and the activity was expressed in arbitrary units based on the highest dilution in which a halo was observed.

### Electron Microscopy

Samples from the MccE492 aggregation assays were placed onto 300-square-mesh copper-rhodium grids coated with carbon and negatively stained with 2% uranyl acetate. Micrographs were taken in a JEOL 1200EX microscope with a tungsten filament operated at 80 kV and with a 50,000X magnification.

## Results

### Microcin E492 forms Intracellular Amyloid Aggregates in *E. coli* Cells

**FIGURE 2:**
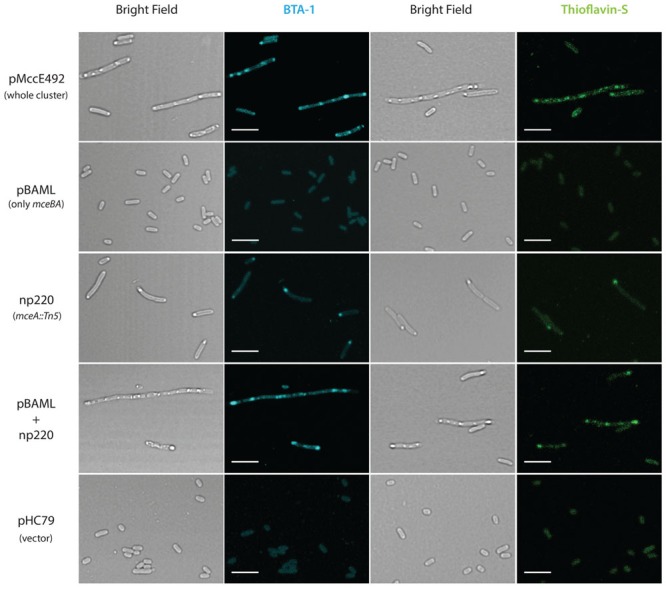
**Microcin E492 accumulates as cytoplasmic amyloid inclusions in *Escherichia coli* cells expressing the whole MccE492 genetic cluster.** *E. coli* cells expressing totally or part of the components of the MccE492 cluster, were grown until stationary phase, stained with the amyloidophilic probes ThS or BTA-1, and visualized by confocal microscopy. Scale bar: 5 μm. pHC79 is the vector used in the constructions pMccE492 and np220.

It has been shown that amyloidogenic proteins from different origins, when expressed in *E. coli*, are accumulated as cytoplasmic inclusions of amyloid nature (Fernández-Tresguerres et al., 2010; Dasari et al., 2011; Espargaró et al., 2012). Extracellular MccE492 amyloid formation has been observed under several conditions, but it is not known if there is amyloid aggregation in the cytoplasm. To investigate this possibility, *E. coli* cells carrying three different constructions expressing the whole, or part of the genetic cluster encoding for the production of active MccE492 were grown in M9 minimal medium at 37°C until late-stationary phase. After harvested, cells were fixed with 4% PFA, stained with the probes BTA-1 and ThS that specifically bind to amyloid aggregates, and visualized by confocal microscopy (**Figure 2**). Cells carrying pMccE492, a plasmid that encodes for the whole MccE492 gene cluster that includes not only the structural and immunity genes but all those necessary for export and maturation, contained a variable number of cytoplasmic inclusions that were visible under bright-field imaging, and that were recognized by both amyloidophilic fluorescent probes as round-shaped foci. These inclusions of amyloid nature were heterogeneous in form and size, and were located at distinct positions of the major axis of the cells (not only at the poles), as reported previously for the hyper-amyloidogenic variant of the WH1 domain of RepA protein from *P. aeruginosa* (Fernández-Tresguerres et al., 2010). Unexpectedly, cells carrying pBAML (comprising only the MccE492 and its immunity genes) that do not export MccE492, did not accumulate inclusions, and behaved as *E. coli* cells carrying the pHC79 control vector. Cells carrying np220, a plasmid expressing the whole gene cluster with the exception of the structural gene of MccE492 that is interrupted by Tn5, showed a few cells with single polar inclusions that were recognized by both amyloidophilic probes, indicating that most of the inclusions observed in the wild type construct are produced by the expression of the *mceA* gene. This was corroborated upon complementation of the mutant np220 with the *mceA* gene from pBAML. Cells carrying both plasmids restored the multiple amyloid inclusions phenotype caused by pMccE492 (**Figure 2**).

Interestingly, the accumulation of MccE492 amyloid inclusions is accompanied with a polymorphism in the cell length. There is a minority of cells that form filaments with a length equivalent to the sum of more than 15 normal cells (Supplementary Figure S1) that can harbor up to 20 amyloid foci. This suggests that massive amyloid accumulation could someway interfere with the septation process, although it remains unclear why this occurs only in a minor fraction of the bacterial population.

### Identification of Amino Acid Residues and Regions Potentially Involved in MccE492 Amyloid Formation

**FIGURE 3:**
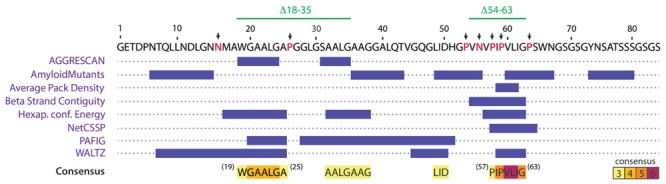
**AMYLPRED2 analysis of MccE492 amino acid sequence revealed putative hydrophobic aggregation hotspots.** The region encompassing residues 57–63 was recognized as pro-amyloidogenic by six different algorithms (highest consensus score in this sequence). A second region (19–25) was recognized with a lower consensus (only four different algorithms). β-strand disruptors (proline) and polar residues flanking or interrupting predicted hotspots (putative aggregation gatekeepers) are shown in red and pointed with black arrows. With this information, a collection of mutants was generated, in which putative gatekeeper residues were individually substituted by alanine, and the two regions comprising putative aggregation hotspots (18–35 and 54–63) were deleted.

One central aspect on the study of amyloid formation is how the primary structure of a protein influences the fibril formation process, and the identification of regions and residues that may act promoting or disfavoring its occurrence. Based on the evaluation of distinct physicochemical properties of its residues, several algorithms have been developed to predict and identify aggregation hotspots inside the primary structure of amyloidogenic proteins (Chiti et al., 2003; Fernández-Escamilla et al., 2004; López de la Paz and Serrano, 2004; Maurer-Stroh et al., 2010; Tsolis et al., 2013). In order to identify putative aggregation hotspots of MccE492, we analyzed its sequence with the AMYLPRED2 web tool, which employs a consensus of 11 different methods and algorithms that predict features related to the formation of amyloid fibrils (Tsolis et al., 2013). Analysis of the MccE492 protein sequence showed that the region comprising residues 57–63 of the processed peptide, especially residues 60–62, has the highest consensus being recognized as pro-amyloidogenic by six different algorithms (**Figure 3**). This region contains several hydrophobic residues (IPVLIG), some of which (VLI) are very commonly found in pro-amyloidogenic regions of several proteins (López de la Paz and Serrano, 2004). Also, inside and near to this region there are several proline residues (P53, P57, P59, P64), which are β-strands disruptors that normally act as “amyloid gatekeepers” diminishing the overall aggregation propensity of the protein (López de la Paz and Serrano, 2004; Beerten et al., 2012). An asparagine residue (N55) was found near to the predicted hydrophobic aggregation hotspot, that due to its polar nature could also act as a gatekeeper. The authors of AMYLPRED2 suggest at least a consensus of five for a reliable prediction. However, the region between residues 20–24 that is recognized only by four algorithms (**Figure 3**) was considered as a secondary amyloidogenic region for the following reasons: first, MccE492 region encompassing residues 16–38 presents 57% of identity and 74% of similarity with the prion PrP protein regions 111–133 (Baeza, 2003); second, this region is also hydrophobic and it is flanked by the putative gatekeeper residues asparagine (N16) and proline (P26). To test these predictions, MccE492 mutants in the residues mentioned above and deletions of the corresponding regions were constructed and its amyloidogenic properties were studied.

### Quantitation of the Proportion of Cells Carrying MccE492 Intracellular Amyloids, Evaluation of Variants with Altered Amyloidogenic Properties, and Identification of Amino Acid Residues Regulating Intracellular Amyloid Formation

Intracellular MccE492 amyloid formation is a phenomenon that can be exploited to implement a fast and simple method for the screening of MccE492 mutants with altered amyloid-formation properties. A comprehensive analysis of these mutants should allow the identification of protein regions controlling the aggregation propensity, providing new insights of how MccE492 amyloid formation occurs. Based on an assay described by Espargaró et al. (2012), we used ThS-staining and flow cytometry analysis to quantitate the proportion of cells harboring cytoplasmic amyloid inclusions upon expression of distinct MccE492 variants. In this way, we were able to measure and compare the amyloid formation propensity of each variant and consequently, to evaluate the relevance of the mutated residue or region in the aggregation process. As a first step, the assay setup required the design and construction of a tightly regulated expression system, which allowed a comparable expression of each MccE492 variant when induced for a defined time lapse. For this purpose, we synthesized the pETAB expression cassette (**Figure 1**, see Materials and Methods) that was ligated to the p33AM plasmid generating p33pETAB. From here, *mceA* gene can be easily replaced by the variants. These constructions were transformed into *E. coli* BL21-AI cells, which expresses the T7 polymerase only after induction with arabinose, therefore MccE492 expression from this system requires the concomitant action of IPTG and arabinose as inductors.

To ascertain that this regulated expression system was working as expected, we tested six random clones of the wild-type form that were induced by incubating them with arabinose and IPTG during 4 h. SDS-PAGE-Immunoblot of total protein extracts showed a prominent band corresponding to MccE492 only in the induced samples (Supplementary Figure S2). In addition, the antibacterial activity of the expressed MccE492 was assessed by co-transformation of the *E. coli* BL21-AI p33pETAB strain with the compatible plasmid np220 (mutated in *mceA*), that provides all the elements required for MccE492 maturation and export. The production of functional active MccE492 of six clones was detected as the presence of growth-inhibition halos over a layer of sensitive bacteria (Supplementary Figure S2). The toxic activity was notably higher in presence of the inducers.

**FIGURE 4:**
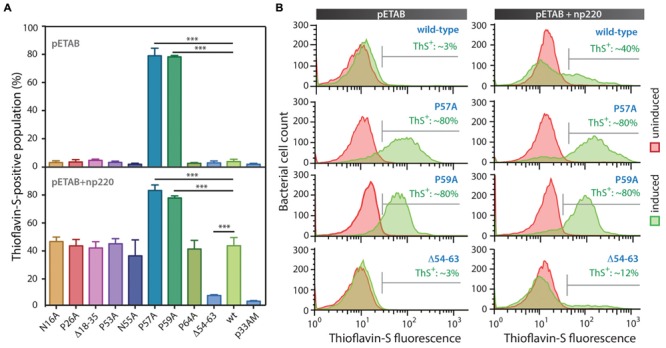
**MccE492 region 54–63 and residues P57 and P59 control *in vivo* amyloid formation.** MccE492 mutants of putative gatekeeper residues or lacking predicted aggregation hotspots were expressed in *E. coli* BL21-AI cells, followed by ThS staining. Bacterial cell populations carrying MccE492 amyloid inclusions (ThS-positive) were quantitated by flow cytometry. **(A)** ThS-positive population frequency upon expression of different MccE492 variants in the absence (top), or co-transformed with np220 (bottom). **(B)** Representative histograms of flow cytometry data obtained for cells expressing wild-type MccE492 or variants with altered aggregation propensity, either in the absence or co-transformed with np220. Error bars show the standard deviation from three independent experiments. ^∗∗∗^*p* < 0.0001. An average of 10000 events were counted per flow cytometry run. p33AM is the vector in which the cassette pETAB was cloned.

Based on the information provided by the *in silico* prediction, we designed a collection of MccE492 variants with substitutions or deletions of specific amino acids. We hypothesized that if the predicted regions act as pro-amyloidogenic stretches, deletion of these regions should diminish aggregation propensity, while substitution of the corresponding putative gatekeeper residues should increase it. Based on this, seven putative gatekeeper residues were individually substituted by alanine (**Figure 3**, black arrows) and two deletion mutants, Δ18–35 and Δ54–63, were generated. All these variants were cloned in p33pETAB and transformed in *E. coli* BL21-AI cells. To evaluate and compare the intracellular aggregation propensity of each variant, their expression was induced for 6 h, and then the cells were stained with ThS and analyzed by flow cytometry. Thus, we determined the proportion of cells carrying amyloid inclusions that were recognized by the dye (**Figure 4**). Expression of wild-type MccE492 led to a very low proportion of cells carrying amyloid inclusions (∼3%), indicating that this protein by itself does not produce a significant amount of amyloid aggregation in the cytoplasm (**Figure 4**, pETAB). This is in agreement with the absence of inclusions observed in cells carrying pBAML, expressing only the MccE492 and its immunity protein (**Figure 2**). Strikingly, variants P57A and P59A formed intracellular amyloids in a very high proportion of cells (∼80%), indicating that substitution of those residues leads to a significant increase in the MccE492 aggregation propensity. This observation shows that both proline residues act as amyloid gatekeepers disfavoring MccE492 amyloid formation. The substitution by alanine of N16, P26, P53, N55, and P65 did not have a significant effect on intracellular amyloid formation, suggesting that these amino acids have a minor or no role controlling MccE492 amyloidogenesis.

Since wild-type protein expression led to a very small amount of ThS-positive cells, at this stage we were not able to evaluate if the deletions of the predicted aggregation hotspots affect MccE492 intracellular aggregation propensity. Considering that more inclusions were observed in cells expressing MccE492 in presence of the other components encoded in the MccE492 genetic cluster (**Figure 2**), the expression of the wild-type MccE492 and its variants was induced in cells carrying np220 (**Figure 4**, pETAB+np220). In this condition, a significantly higher proportion of cells accumulating inclusions upon expression was observed in most of the variants tested. Wild-type MccE492 intracellular amyloid was detected in nearly 40% of the cells, and an indistinguishable behavior was found for all the mutants except for P57A, P59A, and Δ54–63. A significant reduction of aggregation propensity was observed in the variant with the deletion of residues 54–63 (*p* < 0.0001), while the deletion of residues 18–35 had no effect. On the other hand, P57A and P59A mutants maintained the high tendency to form aggregates, although co-transformation with np220 did not increase the proportion of cells accumulating amyloids. This proportion seems to be near the maximum detectable by this method, since no higher frequency was observed in any of the experiments performed, not even with longer induction times (data not shown). The intracellular amyloid formation in these assays was always dependent on the expression of MccE492, since a very low proportion of ThS-positive cells were observed in all the uninduced samples and in the induced cells carrying just the vector p33AM, either alone or co-expressed with np220 (**Figure 4A** and Supplementary Figure S3). This observation suggests that the increase in the frequency of ThS-positive cells caused by np220 co-expression could be due to a synergistic effect between MccE492 and a cluster-encoded factor, and not to an independent aggregation of this putative factor.

**FIGURE 5:**
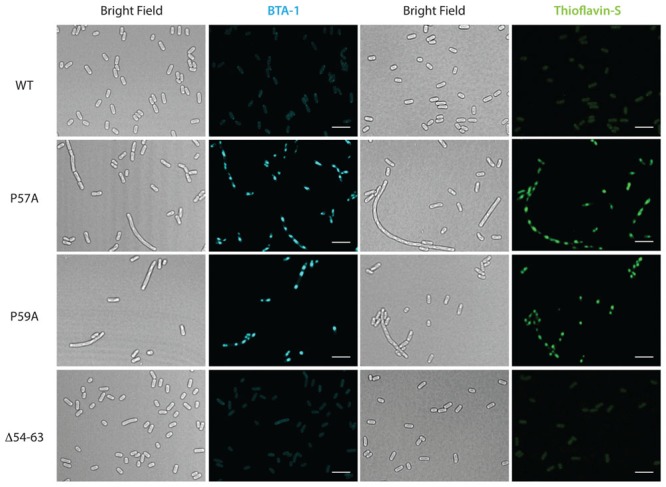
**Formation of amyloid inclusions in *E. coli* cells expressing MccE492 variants with altered aggregation propensity. p33pETAB constructs expressing different variants of MccE492 were transformed into *E. coli* BL21-AI cells.** After 6 h of induction, cells were fixed with PFA, stained with either BTA-1 or ThS and visualized by confocal microscopy. Scale bar: 5 μm.

To further investigate the effect on cell morphology and distribution of the intracellular amyloid detected by ThS-staining and flow cytometry, cells expressing wild type MccE492 from p33pETAB and the identified hypo- and hyper-amyloidogenic variants were induced for 6 h, stained with either BTA-1 or ThS, and visualized by confocal microscopy (**Figure 5**). In agreement with the flow cytometry measurements, cells expressing only wild-type MccE492 from p33pETAB showed none or one very weakly stained intracellular inclusion. A similar situation was observed with Δ54–63, where a faint homogeneous fluorescence was detected upon staining. In contrast, a variable number of inclusions were observed in cells expressing variants P57A and P59A, which showed up under bright-field imaging and were recognized by both amyloidophilic dyes as intense fluorescent foci. These foci were located in different regions/areas of the cells and had different shapes and sizes. Moreover, accumulation of MccE492 inclusions came together with some degree of cell length polymorphism, with a small proportion of cells experimenting a dramatic filamentation and harboring more than 30 amyloid foci (Supplementary Figure S4), as also seen in cells carrying the whole MccE492 production cluster (**Figure 2** and Supplementary Figure S1). As expected, the hyper-amyloidogenic variants as well as the wild-type MccE492 formed amyloid inclusions in cells carrying np220 (Supplementary Figure S5). In contrast, none or one faint polar inclusion per bacteria was observed in cells carrying np220 and expressing the Δ54–63 mutant. Taken together, these results indicate that the region 54–63 of MccE492 has a major role controlling intracellular amyloid formation, encompassing hydrophobic residues that likely form the amyloid core, and two proline β-strand disruptors that act as aggregation gatekeepers. Additionally, at least in the conditions tested, a cluster-encoded factor is likely to act promoting MccE492 intracellular amyloid formation.

### Residues P57 and P59 of MccE492 Control *In vitro* the Kinetics of Amyloid Formation and Loss of Antibacterial Activity

**FIGURE 6:**
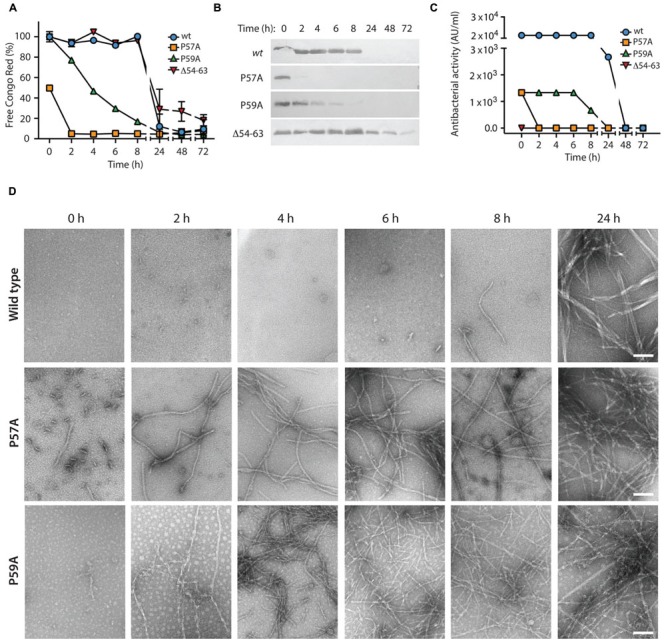
***In vitro* amyloid polymerization of MccE492 variants with altered aggregation propensity.** MccE492 variants (0.4 mg/ml) were incubated in aggregation buffer (see Materials and Methods) and the polymerization progress was evaluated during the time using Congo red binding **(A)**, and visualizing the remaining soluble MccE492 (after centrifuging the samples at 16000 × *g* during 40 min) through SDS-PAGE- Immunoblot **(B)**. In addition, the antibacterial activity was determined at each time **(C)**, and the morphology of the fibrils was studied by negative-stain electron microscopy **(D)**. Error bars show the standard deviation from three independent experiments. Scale bar: 100 nm.

To evaluate if the MccE492 aggregation hotspot and the gatekeeper residues identified in the *in vivo* assays are also relevant for the *in vitro* fibrils formation, we purified wild-type MccE492 and the variants P57A, P59A, and Δ54–63 from the supernatants of induced *E. coli* BL21-AI cells carrying np220 and p33pETAB. Lyophilized MccE492 samples of each variant were dissolved in aggregation buffer to a final concentration of 200 μg/ml and incubated at 37°C with constant shaking for up to 72 h. Amyloid formation kinetics were followed for each variant, monitoring Congo red binding (**Figure 6A**) and determining soluble MccE492 by SDS-PAGE and immunoblotting (**Figure 6B**) at different times. Both Congo red binding and immunoblotting showed that most of the wild-type MccE492 remained soluble until 8 h and was completely aggregated at 24 h. In contrast, P57A and P59A variants aggregated significantly faster. Part of P57A was aggregated at the beginning of the incubation, and was practically completely aggregated at 2 h. The P59A variant also presented a reduced *lag* phase. It began to aggregate at 2 h, and was almost completely aggregated at 8 h, showing a more gradual aggregation kinetics than P57A. On the other hand, the mutant lacking the 54–63 pro-amyloidogenic region aggregated slower than the wild-type MccE492, and soluble protein was still detected even after 72 h of incubation.

Previous studies demonstrated that MccE492 amyloid formation causes loss of antibacterial activity (Bieler et al., 2005; Marcoleta et al., 2013). However, it is not known which MccE492 residues conform the toxic domain, nor if these residues are also important for amyloid formation. To gain further information about this, we tested the antibacterial activity of all the MccE492 mutants generated in this study (Supplementary Figure S6), stabbing colonies of *E. coli* expressing np220 and p33pETAB variants over a layer of sensitive bacteria grown in plates with or without the inducers. Growth inhibition halos were observed in wild-type MccE492, and mutants N16A, P26A, P53A, N55A, P57A, and P59A. On the other hand, Δ54–63, Δ18–23, and P64A lacked antibacterial activity, since no growth inhibition halos were observed. These results indicate that the regions encompassing residues 18–23 and 54–64 are somehow required for the antibacterial activity, while residues N16, P26, P53, N55, P57, and P59 seem to be dispensable.

To evaluate if the increased polymerization propensity of the hyper-amyloidogenic variants correlate with a faster toxin inactivation, we titrated the antibacterial activity during the aggregation assay described above using the critical dilution method (**Figure 6C**). We observed that although both P57A and P59A mutants had antibacterial activity, the initial titer was ∼20-fold lower than wild-type MccE492. As expected, inactivation and polymerization kinetics showed a close correlation, being the loss of the antibacterial activity of the hyperamyloidogenic variants significantly faster.

### The Fibrils formed by Wild-Type MccE492 and the Variants P57A and P59A have a Similar Morphology

*In vivo* and *in vitro* assays showed that P57A and P59A variants present a higher propensity to form fibrils with faster polymerization/inactivation kinetics. However, it is indispensable to know the morphology of the aggregates formed by the variants, to ascertain that they form amyloid fibrils. To this end, samples of the *in vitro* aggregation assay were analyzed by negative-stain TEM (**Figure 6D**). The results were consistent with those observed using Congo red. A few short fibrils and globular aggregates of the wild-type MccE492 were observed at 6 and 8 h, while longer mature fibrils with a width of about 10–12 nm were observed at 24 h, as reported previously (Bieler et al., 2005; Arranz et al., 2012; Marcoleta et al., 2013). On the other hand, the amyloid fibrils formed by both proline mutants presented the same morphology than the wild-type protein, although with an accelerated kinetics of formation. For P57A, short fibrils and globular aggregates were observed even without incubation (time 0), whereas longer mature fibrils were observed at 2 h and in the remaining time points analyzed. In the case of P59A, no fibrils were detected at time 0, but long fibrils were observed after 2 h of incubation (**Figure 6D**).

**FIGURE 7:**
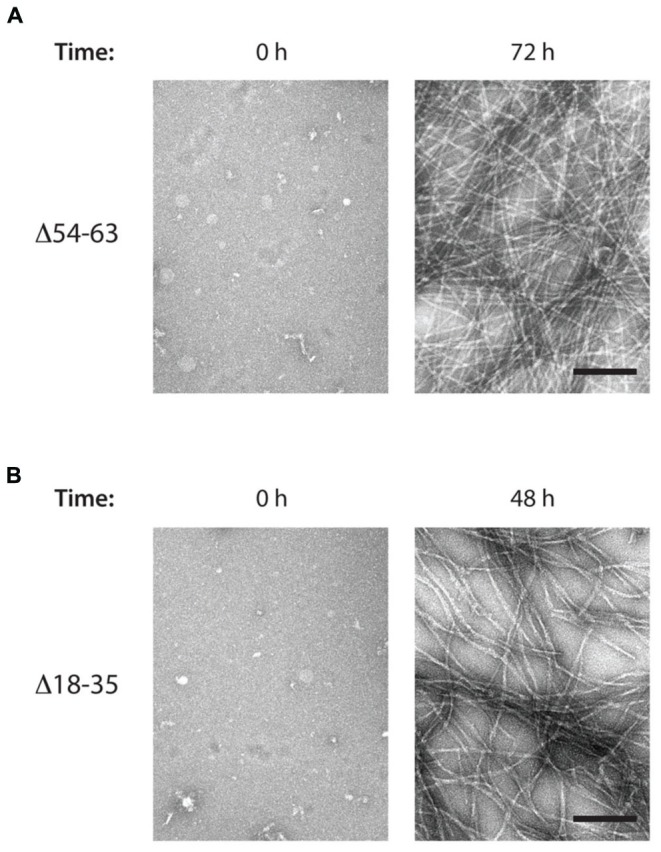
**MccE492 mutants Δ18–35 and Δ54–63 preserve the ability of forming amyloid fibrils *in vitro*.** Negative-stain electron microscopy visualization of amyloid fibers formed by the MccE492 variants Δ54–63 **(A)** or Δ18–35 **(B)**, incubated in aggregation buffer until the indicated time. Scale bar: 100 nm.

Regarding the Δ54–63 mutant, TEM analysis of samples collected at the end of the aggregation assay showed that, despite its decreased aggregation propensity and slower polymerization kinetics, this mutant retains its ability to form fibrils with a similar morphology to the wild-type MccE492 (**Figure 7A**). This suggests that this region is not essential for amyloid formation, and probably there is a secondary aggregation hotspot that could act nucleating amyloid polymerization, although less efficiently. The ability to form amyloid fibrils was also assessed for the mutant Δ18–35, that *in vivo* did not present alterations in amyloid formation. As expected, typical amyloid fibrils although a little more relaxed were formed (**Figure 7B**).

## Discussion

Currently, understanding of amyloid formation is experiencing a change of paradigm. Traditionally, amyloids have been associated with pathological manifestations of neurodegenerative diseases in mammals, but today it is being recognized as a widespread phenomenon with examples from all domains of life (Fowler et al., 2007; Otzen, 2010; Invernizzi et al., 2012). Remarkably, microbes ended up being very successful in controlling and adapting amyloid formation to perform different functions (Hufnagel et al., 2013; Schwartz and Boles, 2013; Romero and Kolter, 2014), although they still remain vulnerable to the deleterious effects of uncontrolled aggregation (Fernández-Tresguerres et al., 2010; Beerten et al., 2012; Gasset-Rosa et al., 2014). MccE492 represents a particular case of what has been defined as a functional amyloid, where the extracellular fibrils formation acts as a mechanism to control the antibacterial activity. In this study, we addressed two major aspects previously unexplored: first, if MccE492 could undergo amyloid aggregation in the cytoplasm of producing cells; and second, which regions or residues are involved in controlling MccE492 amyloid formation.

Our results showed that wild-type MccE492 has a low tendency to form intracellular amyloid, being barely detectable in cells over-expressing this gene. However, its occurrence was highly increased when a plasmid carrying the rest of the MccE492 cluster components was co-transformed, or when MccE492 hyperamyloidogenic variants were expressed. As seen for other amyloidogenic proteins like RepA, cytoplasmic MccE492 amyloid was accumulated as dense inclusion bodies of variable shape, size, and number per cell (Fernández-Tresguerres et al., 2010). This observation was exploited to perform a screening for MccE492 mutants with altered aggregation propensity, establishing that the region comprising residues 57–63, recognized as pro-amyloidogenic by the AMYLPRED2 prediction tool, effectively acts as an aggregation hotspot. Deletion of this region caused a significant decrease in MccE492 intracellular aggregation propensity and the slow down of *in vitro* fibril formation kinetics. The identified aggregation hotspot harbors VLI residues that likely form the β-strand core for amyloid assembly, as demonstrated for other amyloidogenic proteins (López de la Paz and Serrano, 2004). In addition, P57 and P59 residues that are inside this region, probably act as β-strand disruptors, disfavoring MccE492 amyloid formation. Indeed, alanine-substitution of each of these residues dramatically increased MccE492 aggregation propensity *in vivo*, and fastened *in vitro* fibril formation kinetics. The control of amyloid formation by gatekeeper residues seems to be a common strategy for different amyloids, since analysis of *Homo sapiens* and *E. coli* proteomes showed that the positions flanking aggregating stretches are enriched with residues such as lysine, arginine, glutamate, aspartate and proline. Strikingly, 90% of the 26,000 pro-amyloidogenic segments found in the *E. coli* proteome have at least one of these five residues at the first position on either side of the segment (Rousseau et al., 2006). Moreover, gatekeeper residues can be redundant, i.e., they are present several times in the same protein. Thus, aggregating regions from key human proteins such as p53 or huntingtin protein are among the most extensively gate-kept sequences, with a strong enrichment of mutations that disrupt gatekeeper motifs in a set of disease-associated mutations listed in the UniProt database (Reumers et al., 2009). The first four of the abovementioned gatekeeper amino acids are at the bottom of different aggregation propensity scales, mainly as a consequence of their very low hydrophobicity, charge and low β-sheet propensity (Monsellier and Chiti, 2007). Proline, as mentioned previously, is considered a β-strand breaker since its conformational rigidity imposes spatial constraints to the cross-β folding. A more specific role of this residue in regulating amyloid formation has been proposed, where *cis* to *trans* isomerization can act as an intrinsic molecular switch modulating aggregation propensity. It has been shown that the peptidyl prolyl *cis/trans* isomerase cyclophilin A causes a prolongation of the *lag* phase and an increase in the yield and length of fibrils formed by the human amyloidogenic protein stefin B (Smajlović et al., 2009). Additionally, structural and biochemical characterization of early intermediates of β2-microglobulin folding revealed that a *cis* to *trans* isomerization of proline 32 is determinant in the onset of amyloid formation of this protein (Eichner and Radford, 2009), while a similar role of proline isomerization was observed for Ribonuclease A amyloidogenesis (Miller et al., 2010). Understanding the nature of the proline-gatekeeping of MccE492 amyloid formation will provide further information about how this process is regulated and in which circumstances it is favored. The relatively high amount of proline residues found in MccE492 (six), four of them located near to the identified aggregation hotspot, supports the relevance of this amino acid in the modulation of MccE492 amyloidogenesis. It is important to note, however, that the position and sequence context of these residues seems to be determinant, since mutation of only P57 and P59 but not the rest of the proline residues caused a detectable alteration in the aggregation propensity.

Electron microscopy analysis showed that although with a different efficiency, MccE492 mutants P57A, P59A, and even that lacking residues 54–63, are able to form fibrils morphologically similar to those formed by the native protein. This latter observation suggests that an alternative group of residues could act as a pro-amyloidogenic region. One possibly, is the region comprising residues 19–25, which was detected with a lower consensus by the AMYLPRED2 tool. This region also harbors hydrophobic residues flanked by a polar residue (N) and a proline, which qualify as putative aggregation gatekeepers. Additionally, this group of residues is located inside a region (16–37) that has 74% similarity with a portion of the prion-forming domain of the human prion protein (PrP), and also comprises an imperfect repeat of amino acid residues (21-AALGA**P**GG-28 and 32-AALGA**A**GG-39). Curiously, the only different amino acid (in bold) is a proline/alanine, surrounded by non-polar residues. It has been shown that amyloid formation of the CsgA protein (coding major curli subunit) is determined by five imperfect repeats (R1–R5), where only R1 and R5 promote responsiveness to CsgB nucleation and self-seeding by CsgA fibers (Wang and Chapman, 2008; Wang et al., 2008b). Repeats R2–R4 comprise specific aspartic and glycine residues that reduce the aggregation propensity, and thus modulate polymerization efficiency and potential toxicity (Wang et al., 2010). CsgA mutants lacking those gatekeeper residues polymerized *in vitro* significantly faster than wild-type protein, and remarkably, polymerized *in vivo* even in absence of its nucleator CsgB. This points out the possible relevance of the region 19–37 for MccE492 amyloidogenesis. Nevertheless, deletion of residues 18–35 did not affect the intracellular amyloid formartion propensity (**Figure 4**), and the *in vitro* aggregation products were typical amyloid fibrils (**Figure 7**). Also, a mutant carrying substitution P26A (rendering a perfect repeat) showed the same intracellular aggregation behavior than the wild-type protein. A similar situation was observed when the alanine 37 was substituted by a proline (data not shown), arguing against the significance of the repeats. In spite of the evidence abovementioned, it cannot be excluded that this region may act leading MccE492 amyloid formation in the absence of the primary aggregation hotspot. Taken together, these results point out the very robust amyloidogenic capacity of MccE492, which is kept even after deleting 18 out of 42 residues of its N-terminal half, or at least 10 out of 42 residues of its C-terminal half.

In agreement with previous reports, MccE492 amyloid formation correlated with the loss of antibacterial activity (Bieler et al., 2005; Marcoleta et al., 2013). The hyperamyloidogenic variants P57A and P59A retained the antibacterial activity but displayed significantly faster inactivation kinetics (**Figure 6** and Supplementary Figure S6). This observation suggests that these residues are dispensable for the pore-forming activity, and that through these mutations it is possible to modulate how long the toxin remains active. From this, it is plausible that alternative substitutions of the gatekeeper residues could generate a battery of toxin variants with different inactivation kinetics.

We found that MccE492 intracellular amyloid formation was observed in three circumstances: when the whole MccE492 genetic system was expressed; when expressing the hyperamyloidogenic mutants P57A and P59A from the pETAB cassette; and when the wild-type form was expressed from the abovementioned cassette in the presence of np220, a plasmid expressing the genetic determinants encoded in the MccE492 cluster with the exception of the structural gene. These observations suggest that a cluster-encoded factor could act nucleating or promoting MccE492 amyloid formation *in vivo*. Although at first sight this result may appear as unexpected, the general behavior of MccE492 amyloid formation is similar to the well-characterized curli amyloid system, for the following reasons: first, extracellular *in vivo* amyloid formation by CsgA requires an amyloid minor component, the nucleator CsgB (Chapman et al., 2002; Hammer et al., 2007), so it is perfectly plausible that MccE492 as well may require the presence of a nucleator for *in vivo* amyloid formation; second, both purified CsgA and MccE492 can form amyloids *in vitro* with the typical kinetics that has a *lag*, growth and stationary phase (Bieler et al., 2005; Wang et al., 2007). The requirements for amyloid formation *in vitro* seems to be less restrictive because there is no need of another component, probably because the use of high protein concentrations overcomes the requisite of a nucleator; and third, the duration of the *lag* phase of both proteins can be significantly shortened *in vitro* by the addition of sonicated preformed fibrils (Wang et al., 2007; Marcoleta et al., 2013). In the same line, the origin of the few single polar inclusions observed in cells carrying np220 could be explained by the aggregation of a factor such as CsgB, that also has amyloid properties (Hammer et al., 2007). Currently, we are working on the identification of the putative nucleator factor.

Whether intracellular MccE492 aggregation has a physiological role, and how its occurrence affects the cellular metabolism, are issues that have to be investigated. Regarding the latter, there are examples indicating that the accumulation of amyloid inclusion bodies are toxic for *E. coli* cells, as seen after overexpression of hyperamyloidogenic variants of RepA and CsgA (Fernández-Tresguerres et al., 2010; Wang et al., 2010). Moreover, it was shown that the identity of residues flanking an aggregation prone region (σ32β) fused to GFP had a significant effect on bacterial growth, where fusions harboring σ32β flanked by its natural gatekeepers displayed the greatest competitive fitness (Beerten et al., 2012). The impact of intracellular amyloid accumulation over cellular functions makes necessary the existence of cellular mechanisms controlling its occurrence. In this respect, chaperone machineries seem to play an important role. In the case of the RepA-WH1(A31V) prionoid, it was shown that DnaK but not ClpB participates in the remodeling of amyloid inclusions, controlling the transition between mild toxic comet-shaped aggregates and highly toxic globular particles (Gasset-Rosa et al., 2014). In contrast, the disaggregase activity of the ClpB chaperone is required to propagate the yeast prion [PSI^+^] in *E. coli* cells, probably by means of fragmenting higher order intracellular amyloid aggregates to generate smaller seed particles (propagons) that can be inherited to daughter cells during cell division (Yuan et al., 2014). This apparently controversial role of ClpB chaperone in modulating amyloid formation in *E. coli* cells points out that amyloids formed of proteins from different origins not necessarily are subject of the same control mechanisms, even when they are expressed in the same host. Additionally, it has been demonstrated for curli that an efficient secretion system and chaperone network ensures that CsgA does not form intracellular amyloid aggregates. Moreover, amyloid formation in the periplasm is prevented by the CsgC protein, which selectively inhibited aggregation of CsgA and also α-synuclein (Evans and Chapman, 2014; Evans et al., 2015). We are currently working in establishing the impact of MccE492 intracellular amyloid formation on the cellular metabolism, and the potential role of chaperones or other factors in controlling this phenomenon. In this regard, preliminary observations show that MccE492 intracellular amyloid accumulation does not seem to have the highly toxic effects observed for other amyloids such as the RepA-WH1(A31V) prionoid. This suggests that MccE492 intracellular amyloid formation may have a role sequestering potentially harmful soluble or oligomeric forms, operating in a similar way as the extracellular toxin inactivation process. In addition, the fact that wild-type amyloid inclusions are observed only when the whole genetic cluster of MccE492 is expressed further support the notion that this phenomenon may have a physiological role. It is important to point out that formation of intracellular amyloid inclusions seems to be a dynamic process, because part of the expressed protein is exported, even in the case of the hyperamyloidogenic mutants. Thus, the formation of amyloid inclusions could be the consequence of MccE492 accumulation because of a limited exporting capacity. Also, although MccE492 expressed from the whole cluster context have a high tendency to form intracellular amyloid inclusions, it could be possible that other factors like chaperones ensure solubility for exporting a proportion of the produced bacteriocin.

One further aspect to be considered is if the immunity protein MceB plays any role in MccE492 intracellular amyloidogenesis. Since the expression of MccE492 in the absence of its immunity protein is lethal to the cell (Bieler et al., 2006), both proteins had to be co-expressed in all the experiments, hampering the possibility to directly compare MccE492 amyloid formation in presence or absence of MceB. However, we believe that it is very unlikely that this protein participates in the amyloidogenic process, because MceB is an integral membrane protein with three transmembrane helixes and not detected in the cytoplasm (Lagos et al., 1999), therefore the neutralization of MccE492 by MceB does not occurs in this compartment. Although the exact mechanism by which the immunity protein neutralizes the MccE492 pore-forming activity is unknown, the interaction would occur at the moment that MccE492 is inserted into the inner membrane preventing the correct insertion to form the pore. On the other hand, this protein does not form inclusions by itself, because when co-expressed with the wild type or hypo-amyloidogenic mutants no inclusions were observed.

MccE492 has advantageous properties for its use as a model to understand amyloid formation and its consequences. First, it is a small protein (84 amino acids), which allows the study of its amyloid behavior using variants of the full peptide, and not only regions or domains of the protein related with amyloid formation. Second, it polymerizes in a shorter time scale than other amyloidogenic proteins, and finally, it can be easily purified from culture supernatants. These properties, and the use of the intracellular amyloid formation phenomenon to screen for defects in amyloid formation, constitute a model useful to study the effect of extrinsic and cellular factors involved in the amyloidogenesis of this and possibly other proteins that can be expressed in *E. coli*.

## Author Contributions

AM, OM, RL conceived the work. PA, AM, PL-R, RA, JMV, OM, RL designed the experiments, analyzed the data and interpreted the results. PA, AM, PL-R, RA, JMV conducted the experiments. PA, AM, RL wrote the manuscript. OM and JMV critically revised the manuscript. All the authors approved the final version of the manuscript.

## Conflict of Interest Statement

The authors declare that the research was conducted in the absence of any commercial or financial relationships that could be construed as a potential conflict of interest.

## Abbreviations

BTA-1: 2-4′-methylaminophenyl benzothiazole
MccE492: microcin E492
PBS: phosphate-buffered saline
PFA: paraformaldehyde
TCA: trichloroacetic acid
ThS: thioflavin S
