# Supplementary material for: Identification of Key Amino Acid Residues Modulating Intracellular and In vitro Microcin E492 Amyloid Formation
Source: Front Microbiol. 2016 Jan 28;7:35. doi: 10.3389/fmicb.2016.00035 (PMC4729943; doi:10.3389/fmicb.2016.00035)
Supplement: Supplementary file 1 [file Data_Sheet_1.PDF]

## SUPPLEMENTARY MATERIAL

# Identification of key amino acid residues modulating intracellular and *in vitro* microcin E492 amyloid formation

Paulina Aguilera, Andrés Marcoleta*, Pablo Lobos-Ruiz, Rocío Arranz, José María Valpuesta, Octavio Monasterio , Rosalba Lagos*

Correspondence: Rosalba Lagos: rolagos@uchile.cl
Andrés Marcoleta: amarcoleta@uchile.cl

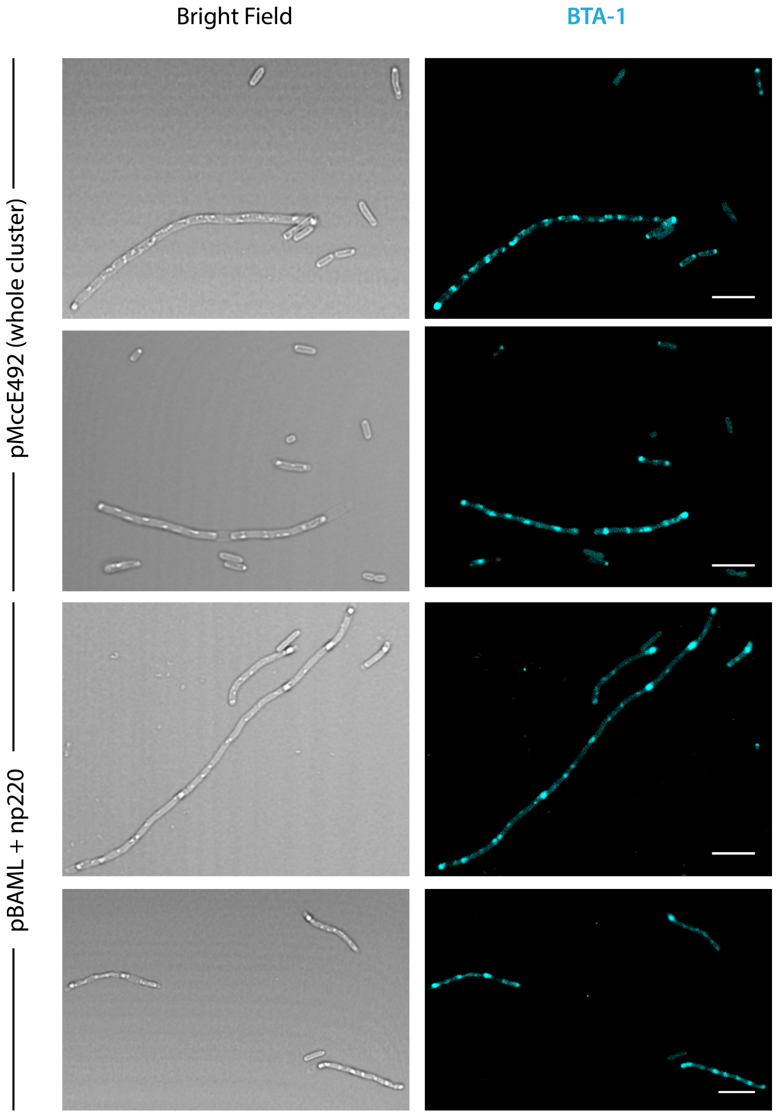


**FIGURE S1. Amyloid inclusions accumulation in *E. coli* cells carrying the whole MccE492 production cluster is accompanied by a polymorphism in cell length.** Cells carrying pMccE492 or both np220 and pBAML were grown in M9 medium until late-stationary phase and then were PFA-fixed, stained with BTA-1 and visualized by confocal microscopy. Scale bar: 5 µm.

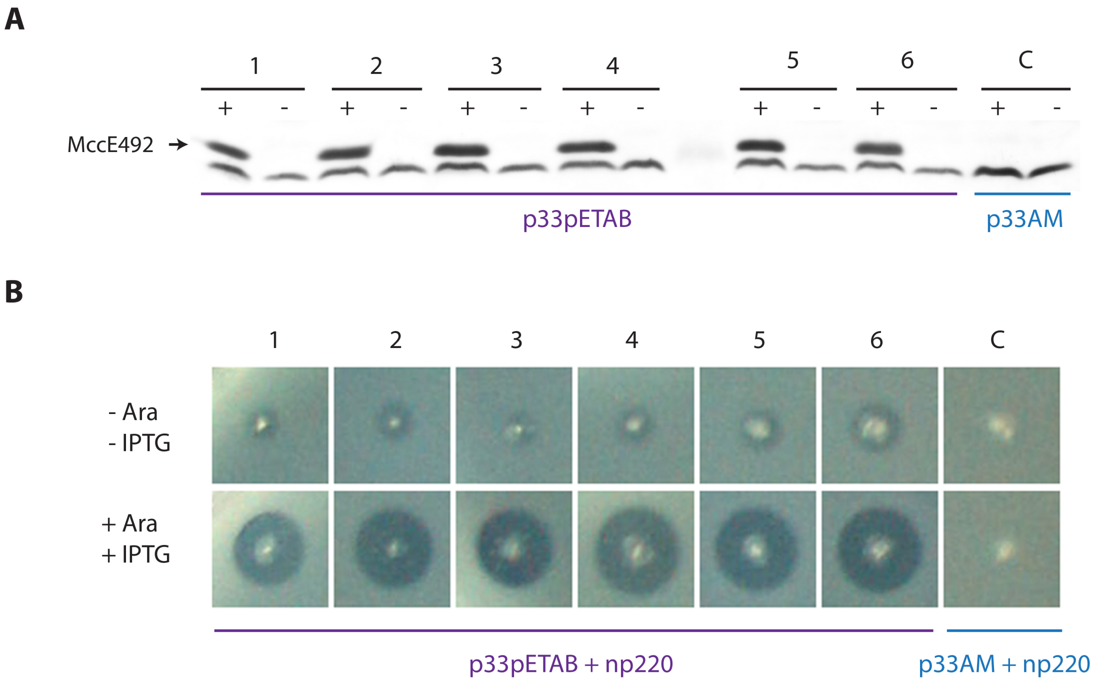


**FIGURE S2. Expression of MccE492 from the pETAB cassette.** (A) Immunoblot of SDS-PAGE of protein extracts prepared from six different clones harboring p33pETAB, grown in presence (+) or absence (-) of IPTG and arabinose. A clone carrying p33AM was also included as a negative control, and an anti-MccE492 polyclonal antibody was used. The *E. coli* extract presented a low-molecular mass protein that was recognized by the MccE492 polyclonal antibody. This protein is unrelated to the MccE492 system because it was present in the control transformed with the vector used for the constructions and in both, induced and uninduced conditions. (B) p33pETAB was transformed into *E. coli* BL21-AI cells carrying np220, and six transformants were stabbed over a lawn of bacteria sensitive to MccE492. Production and export of active MccE492 from the combination of p33pETAB and np220 was confirmed by the detection of growth-inhibition halos resulting from the six stabbed clones, that were not observed in a clone carrying np220 plus p33AM (negative control, C). The antibacterial activity was higher in the induced samples carrying the expression cassette.

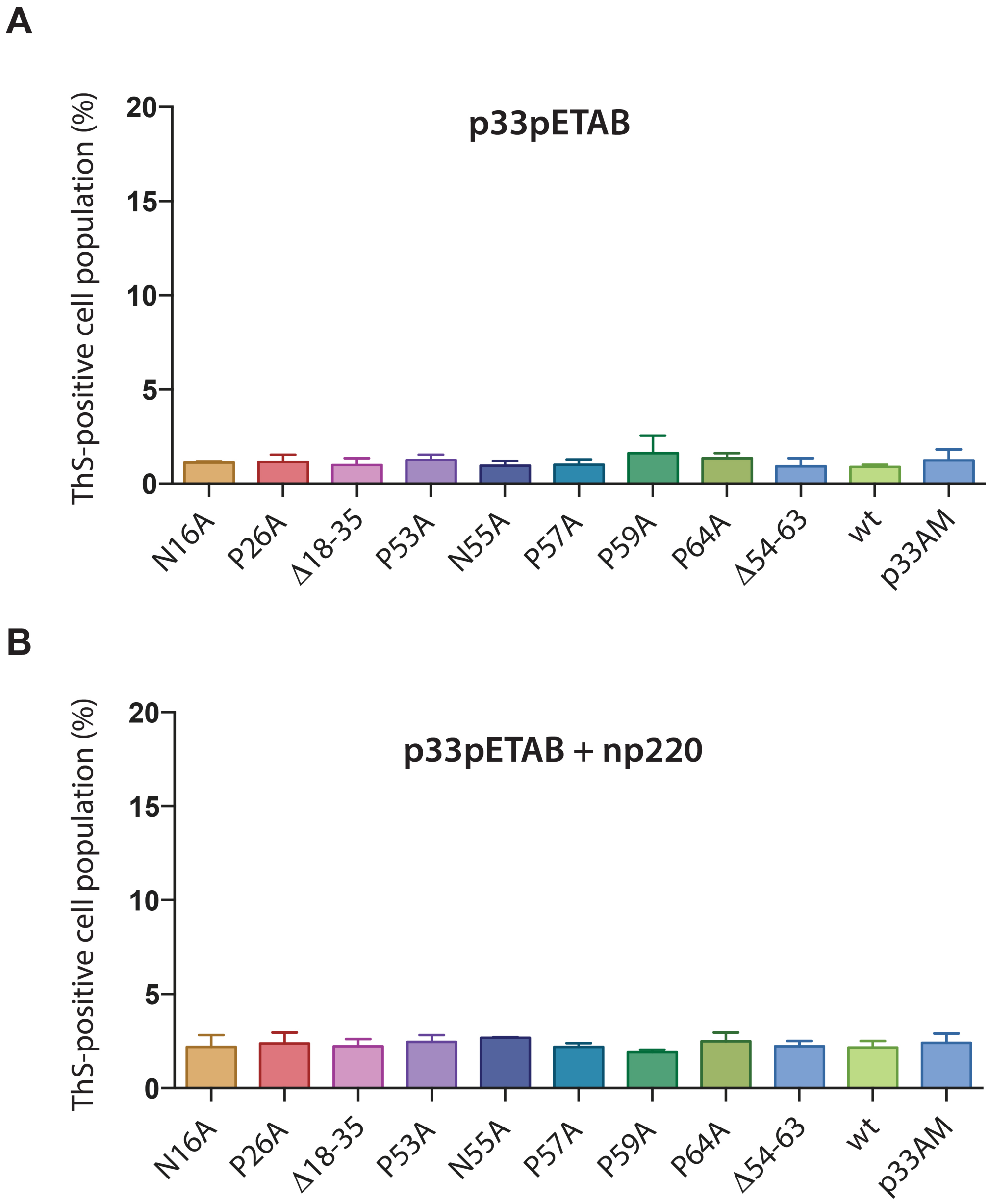


**FIGURE S3. MccE492 intracellular amyloid formation is almost negligible in uninduced cells harboring p33pETAB expression cassette.** MccE492 mutants of putative gatekeeper residues or lacking predicted aggregation hotspots were expressed in *E. coli* BL21-AI cells, followed by ThS staining. Bacterial cell population carrying MccE492 amyloid inclusions (ThS-positive) was quantitated by flow cytometry. Histograms show the ThS-positive population frequency in uninduced samples of different MccE492 variants without (A), or co-transformed with np220 (B). Error bars show the standard deviation from three independent experiments. An average of 10000 events were counted per flow cytometry run.

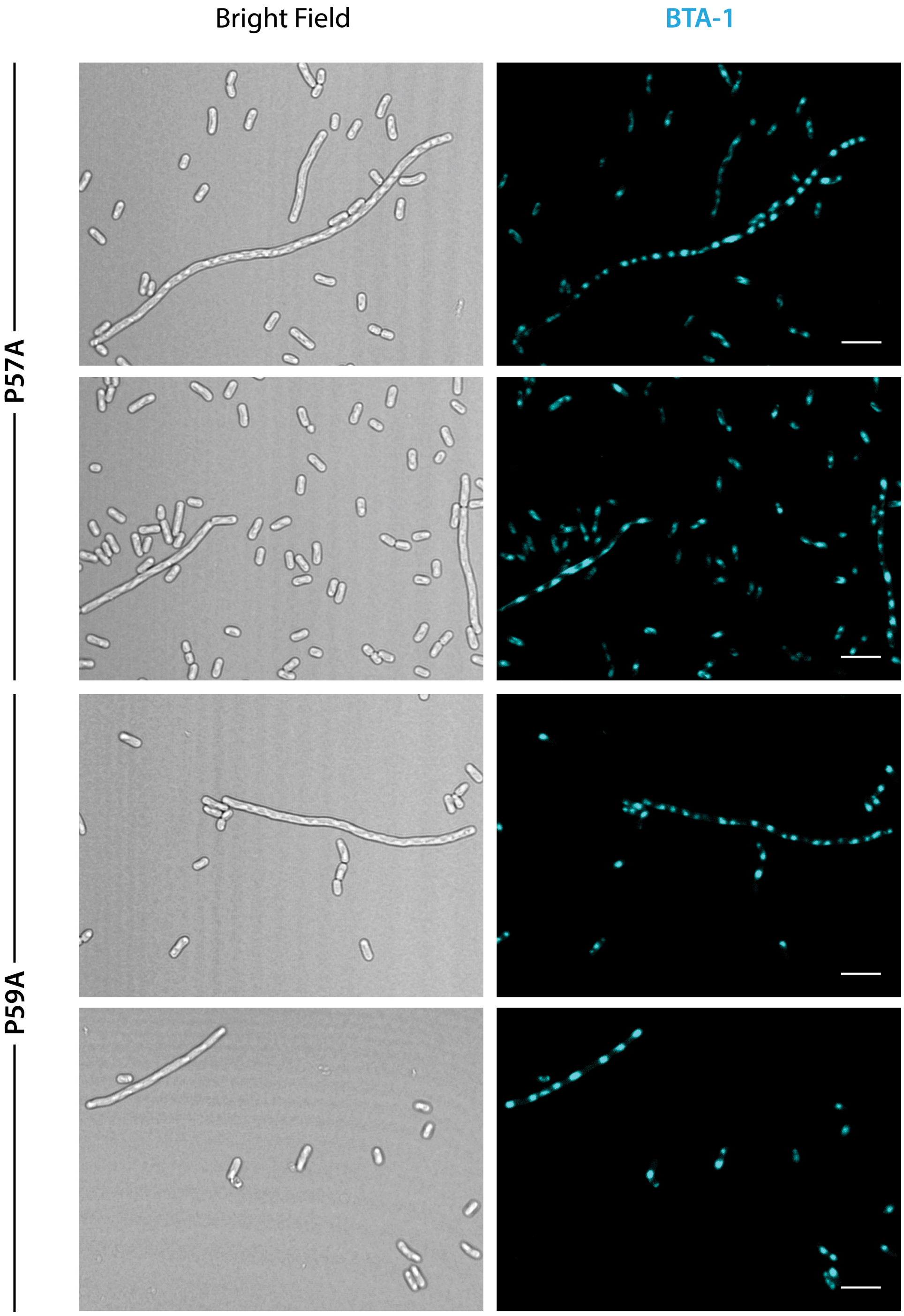


**FIGURE S4. Amyloid inclusions accumulation of the hyper-amyloidogenic P57A and P59A variants is accompanied by cell length polymorphism.** p33pETAB constructs expressing the MccE492 variants P57A or P59A were transformed into *E. coli* BL21-AI cells. After 6 h of induction, cells were fixed with PFA, stained with BTA-1 and visualized by confocal microscopy. The pictures show cell filamentation and massive intracellular amyloid accumulation. This phenotype was observed in a small part of the cell population. Scale bar: 5 μm.

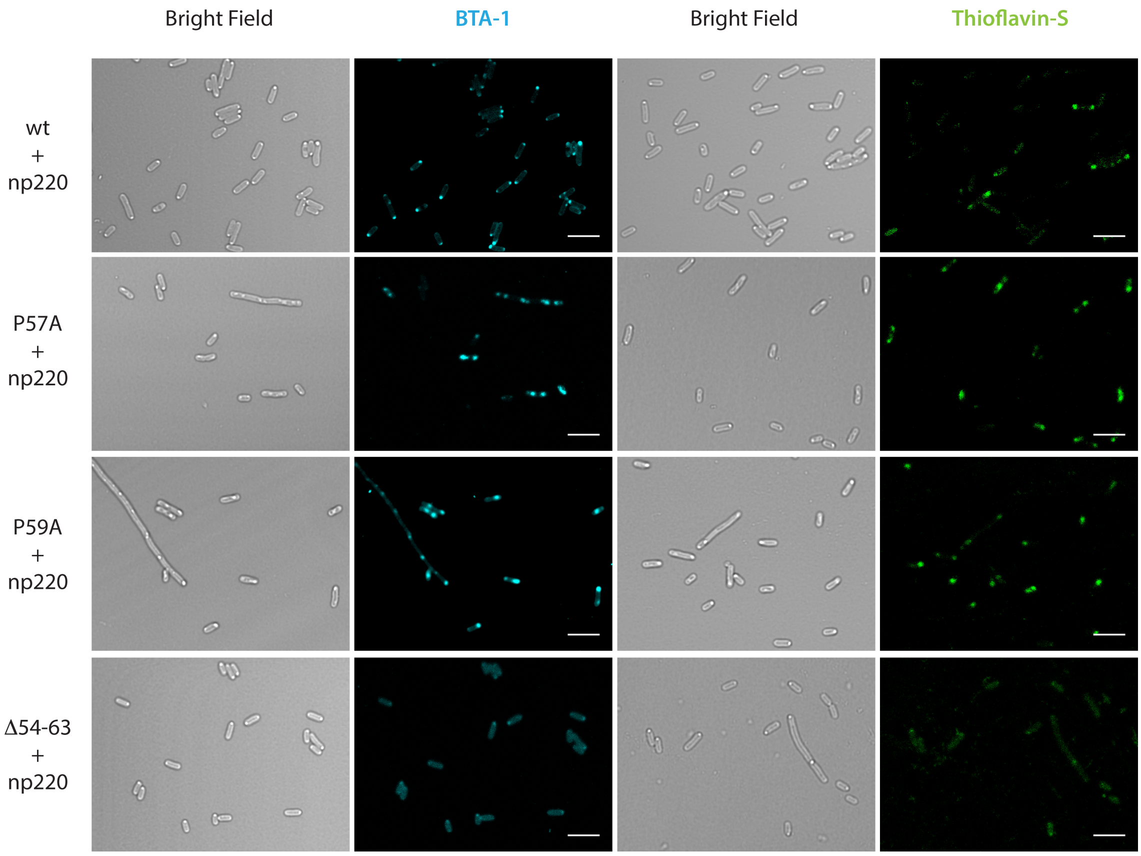


**FIGURE S5. Intracellular amyloid formation upon expression of different MccE492 mutants in cells carrying np220.** p33pETAB constructs expressing MccE492 variants with altered aggregation propensity were transformed into *E. coli* BL21-AI/np220 cells. After 6 h of induction, cells were fixed with PFA, stained with either BTA-1 or ThS and visualized by confocal microscopy. Scale bar: 5 μm.

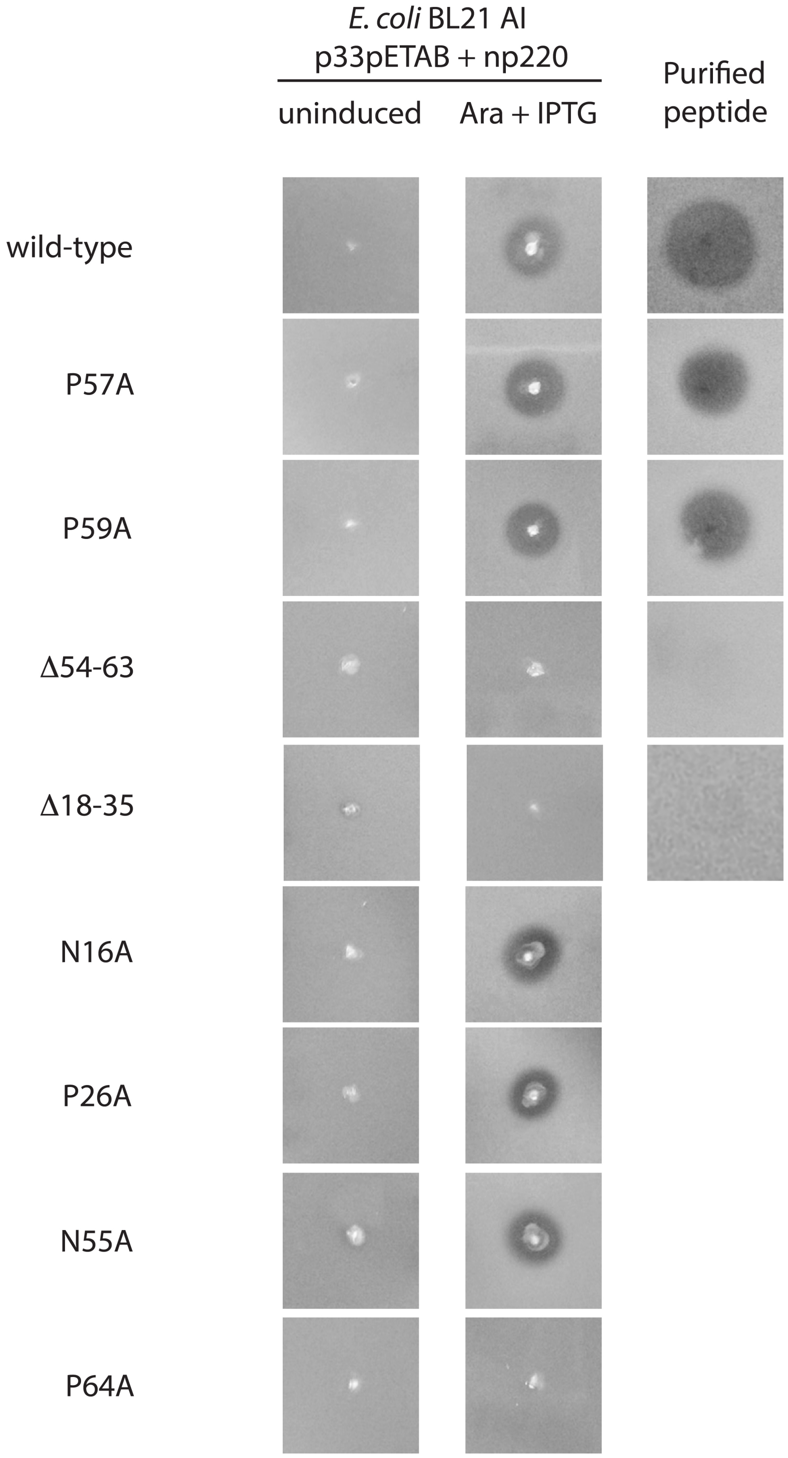


**FIGURE S6. Antibacterial activity of the MccE492 mutants constructed in this study.** Colonies from *E. coli* BL21AI/np220 cells producing distinct MccE492 variants were stabbed into a layer of sensitive bacteria. Antibacterial activity was detected by the formation of growth inhibition halos. The activity of purified MccE492 used for the *in vitro* assays was tested depositing a drop of ACN fractions obtained after the purification process (see Materials and Methods).
